# Paper-based upconversion fluorescence resonance energy transfer biosensor for sensitive detection of multiple cancer biomarkers

**DOI:** 10.1038/srep23406

**Published:** 2016-03-22

**Authors:** Sai Xu, Biao Dong, Donglei Zhou, Ze Yin, Shaobo Cui, Wen Xu, Baojiu Chen, Hongwei Song

**Affiliations:** 1 State Key Laboratory on Integrated Optoelectronics, College of Electronic Science and Engineering, Jilin University, Changchun 130012, People’s Republic of China; 2 Department of Physics, Dalian Maritime University, Dalian 116026, People’s Republic of China

## Abstract

A paper-based upconversion fluorescence resonance energy transfer assay device is proposed for sensitive detection of CEA. The device is fabricated on a normal filter paper with simple nano-printing method. Upconversion nanoparticles tagged with specific antibodies are printed to the test zones on the test paper, followed by the introduction of assay antigen. Upconversion fluorescence measurements are directly conducted on the test zones after the antigen-to-antibody reactions. Furthermore, a multi-channel test paper for simultaneous detection of multiple cancer biomarkers was established by the same method and obtained positive results. The device showed high anti-interfere, stability, reproducible and low detection limit (0.89 ng/mL), moreover it is very easy to fabricate and operate, which is a promising prospect for a clinical point-of-care test.

Cancer biomarker is a significant indicator to establish the presence of a disease or to monitor the therapeutic effect of a drug. Among them, carcinoembryonic antigen (CEA) is a commonly used cancer biomarker associated with many human cancers, including colorectal, pancreatic, gastric carcinoma^1^,^2^. Moreover, CEA is sensitive to cancer recurrence, thus, it is often used to determine the efficacy of cancer therapy, monitor the disease progression and prognosis, etc. Many efforts have been made to develop new technologies to improve the sensitivity for cancer biomarker analysis^3^,^4^,^5^. Currently, most methods are performed under laboratory conditions, commonly requiring expensive instruments and trained personnel. Thus, the development of novel methods for cancer antigen detection is very necessary. The paper-based analytical device (PAD), which is a potential portable diagnostic technology was present by Whitesides in 2007^6^. Since then, more and more attentions have been paid to PAD because of its advantages including low cost, facile use, and so on^7^. Recently, kinds of PAD have been developed in the detection of various substances including enzyme, cancer antigen, and DNA^8^,^9^,^10^,^11^. Fluorescent detection is very popular in the paper-based assay field owing to its higher sensitivity. However, conventional fluorescent materials such as fluorescin, organic dyes and quantum dots, are usually excited by high-energy ultraviolet (UV) or visible (vis) light, unfortunately, UV-vis excitation often results in autoflorescence and light scattering on the paper substrate^12^. Lanthanide (Ln^3+^)-doped upconversion nanoparticles (UCNPs) can be considered as a good alternative as a fluorescence biolabel. Previous researchers have reported UCNPs can avoid the autofluorescence and scattering light caused by UV-vis excitation due to its NIR-excitation nature, which considerably improves the sensitivity and photostability of fluorescence probes^13^,^14^,^15^. In view of the high complexity of clinical specimens and paper-based substrates, UCNPs would be very suitable for PAD analytics^8^,^9^. In the field of upconversion fluorescence sensing, fluorescence energy transfer (FRET), which is a nonradiative process characterized by energy transfer between an excited donor and an acceptor through long-range dipole-dipole interaction, is widely used as a spectroscopic principle in the detection of ions, DNA, cancer biomarkers, and so on^16^,^17^,^18^.

However, it is well known that no single cancer biomarker is specific and sensitive enough to meet the strict diagnostic demand, single cancer biomarker detection is often easy to cause false positives and negatives result, thus, simultaneous detection of multiplex cancer biomarkers related to a certain cancer is very important to achieve reliable diagnosis of cancer^19^. Compared with the traditional single analyte assay, the simultaneous multiple analytes assay exhibited a lot of advantages such as short analytical time, small sample volume and high accuracy^20^,^21^. In this work, a novel upconversion test paper is obtained by printing UCNPs on the paper substrate using nano-printing technology for CEA detection. Figure 1 presented the procedure of the PAD based on the FRET using UCNPs as donors. Furthermore, a detection array is also established for multi-analytes simultaneous detection. The combination of PAD with UCNPs for simultaneous detection of various cancer biomarkers affords a potential technique for portable detection with expected simplicity, accuracy, and sensitivity, promoting the fluorescence detection applied in the clinical diagnosis.

## Results and Discussions

### Characterization of PEI-NaYF_4_:Yb,Tm UCNPs and conjugation of UCNPs with CEA antibody

For biodetection application, the nanoparticles must be hydrophilic, the UCNPs commonly used were synthesized in oleic acid and 1-octadecene, which are hydrophobic organic solvents and are not water-soluble due to the lack of hydrophilic functional groups on their surfaces. Typically, additional surface-modification steps are required to link appropriate functional groups (e.g. COOH, NH_2_, or SH) to obtain a hydrophilic surface for further conjugation of biomolecules^22^,^23^,^24^. The complicated procedures may affect the morphology and self-luminescence intensity of UCNPs, and this is disadvantage for potential applications of biodetection^25^. Considering of these points, we adopted one-step solvothermal route to prepare amine-functionalized NaYF_4_: Yb/Tm UCNPs with PEI as surfactant that anchors on the surface of the nanoparticles for subsequent conjugation with CEA antigen.

TEM graph of spherical morphology of the PEI-NaYF_4_: Yb,Tm UCNPs before and after CEA adsorption were shown in Fig. 2(a,b), respectively. It can be seen that the nanoparticles before and after CEA adsorption are both uniform in morphology and with an average diameter of 16 nm. Fig. 2(c) exhibits XRD patterns of the UCNPs. It can be seen that all peak positions and intensities can be well indexed in accordance with cubic NaYF_4_ crystals (JCPDS: 77-2042), suggesting high product crystallinity. PEI as capping agent can control the growth of UCNPs and render them a positive ζ potential of +20.6 mV in the colloidal solution, CEA antibody is negatively charged in the carbonate buffer as the isoelectric point of CEA antibody is 5.0^26^, which is lower than the pH of the buffer, and thus, it can conjugate with UCNPs through electrostatic interaction. The adhesion efficiency of CEA is calculated as 87.66%. (The detail information was shown in Fig. S2) After conjugating of UCNPs and CEA antibody, the ζ potential of nanoparticles shifts from +20.6 mV to +11.5 mV. To verify the conjugation of the UCNPs and the antibody, the absorption spectra of the UCNPs, CEA antibody and the CEA antibody-UCNPs coalition were measured. The absorption of antibody at 280 nm was clearly observed in the spectrum of CEA antibody-UCNPs coalition. (Fig. S3)

### Fluorescence response characteristics of FRET PAD for CEA detection

**Figure 1:**
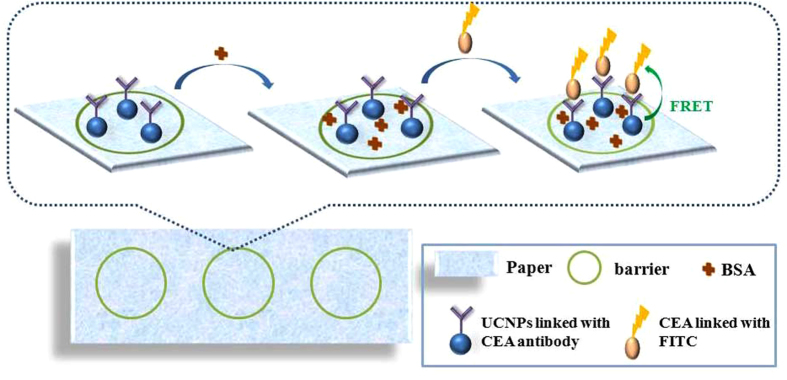
Procedure of the PAD based on the FRET using UCNPs as donors and FITC as acceptor.

**Figure 2:**
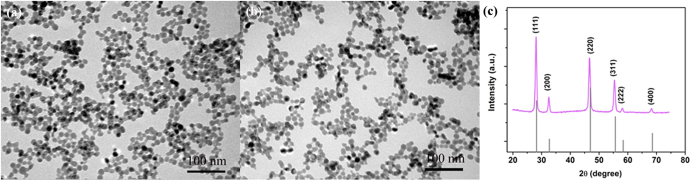
(**a**) TEM of as-prepared PEI-NaYF_4_:Yb,Tm nanoparticles (**b**) TEM of PEI-NaYF_4_:Yb,Tm nanoparticles after adsorption of CEA; (**c**) XRD patterns of the NaYF_4_:Yb,Tm UCNPs in contrast to the standard card for cubic phase NaYF_4_.

**Figure 3:**
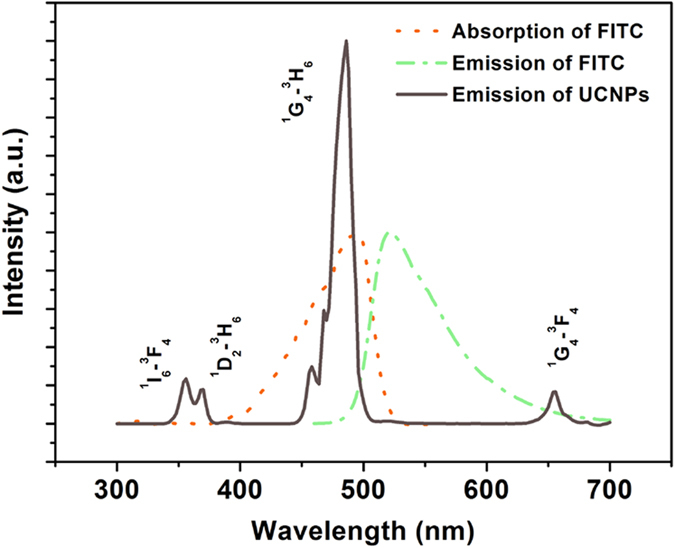
Emission (grey line) spectrum of NaYF_4_:Yb,Tm UCNPs; absorption (orange dashed line) and emission (green dashed line) spectra of FITC.

The PAD was fabricated by nano-printing technology, briefly, the nanoparticles (CuS nanoparticles, UCNPs) solutions were injected into cartridges, and then printed on the surface of a piece of filter paper with a HP commercial printer^27^. We firstly drew a circle with diameter of 1 cm on the computer remotely, and then the paper was printed with hydrophobic CuS nanoparticles according with the graph on the computer as a hydrophobic wall to isolate test zones contained hydrophilic centers, finally, the diameter of the zone was determined with a ruler. The hydrophobic barrier ensured that aqueous drops applied to the paper would remain localized in defined reaction zones, providing opportunity to achieve the low volume desired for the detection. Figure S4 showed the digital photo of the test paper. The procedure of the PAD based on the FRET using UCNPs as donors was presented in Fig. 1. First, the CEA antibody-UCNPs coalition was printed on the test zone as a fluorescence probe, this was followed by 5 μL 5% bovine serum albumin (BSA) solution dropping on the test zone as block, then the paper was dried at 37 ^o^C for 30 min. SEM of UCNPs printing on the test paper was shown in Fig. S5, it can be seen that UCNPs adhered to the surface of the paper and aggregated to some extent after printing on test zone. Different concentration of FITC labeled CEA antigen solution was dropped on the specific test zone, following by softly washing with PBS solution. Subsequently, the paper was scanned with a photomultiplier combined with a monochromator, a continuous 980 nm diode laser was used to pump the samples to investigate the steady-state spectra. Here FITC was selected as an energy acceptor to couple with the NaYF_4_:Yb,Tm UCNPs, because it has a broad excitation band centering at 490 nm which can well overlap with the ^1^G_4_-^3^H_6_ emission lines of Tm^3+^ peaking at 484 nm, as is shown in Fig. 3. Another important reason for FITC being chosen as the FRET acceptor is that the UCNPs can be excited using a NIR laser at 980 nm where FITC cannot be photoexcited, thereby avoiding FITC excitation by external light and eliminating luminescence background interference. Excitation of NaYF_4_:Yb^3+^,Tm^3+^ UCNPs triggers energy transfer (ET) from Tm^3+^ to FITC within a given proximity through reaction between CEA antigen and antibody, and results in emission from FITC at its characteristic wavelength.

**Figure 4:**
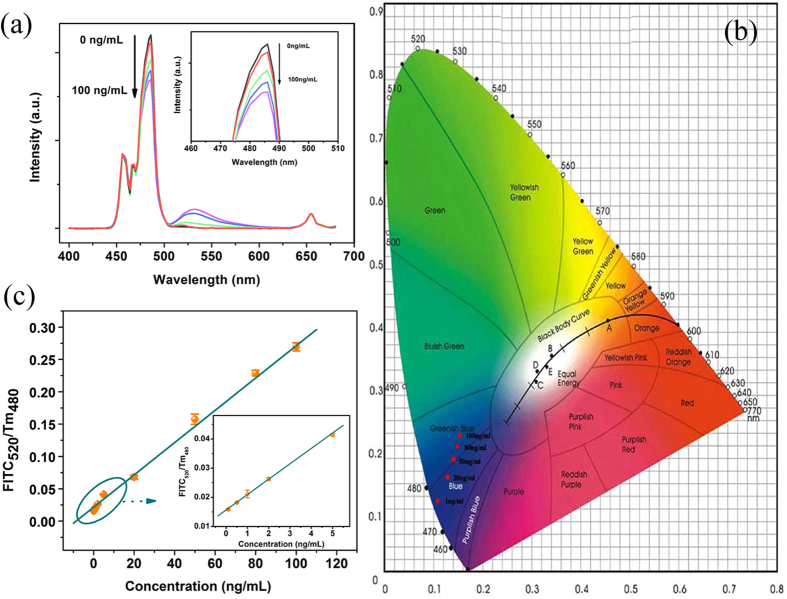
(**a**) FRET spectra of PAD at different concentrations of CEA (inset: enlarged portion of the curve peaks); (**b**) CIE chromaticity coordinates for test zones with different concentration of CEA; (**c**) Calibration. curve of FRET detection on PAD for the integrated luminescence intensity ratio FITC_520_/Tm_480_ versus the concentration of CEA.

**Figure 5:**
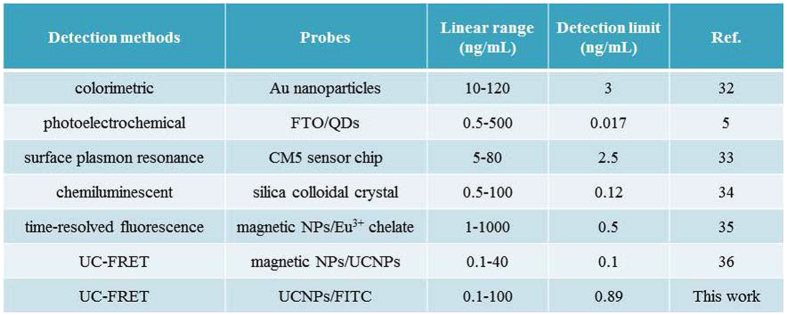
CEA limit of detection (LOD) and linear range of seven detection methods.

As shown in Fig. 4(a), upon excitation of UCNPs under 980 nm laser diode, the emission band of FITC centered at 520 nm (referred as FITC_520_ here after) gradually increases with the increasing amount of CEA antigen, accompanying by the expense of ^1^G_4_-^3^H_6_ emission band of Tm^3+^ centered at 480 nm (referred as Tm_480_ here after), the emission intensity of 653 nm almost unchanges. The inset showed the enlarged portion of the curve peaks ranging from 460 nm–510 nm. CIE chromaticity is a diagram represented by the nominal value, which x and y represent the red and green component, respectively, and the values on the border indicate the wavelength of the spectrum. All the actual nature colors are located within the closed curve. The CIE chromaticity coordinates for test zones with different concentration of CEA are shown in Fig. 4(b), the color of the emission light gradually changed from blue to greenish blue with the increasing concentration of the CEA from 1 to 100 ng/mL. Fig. 4(c) shows the curve of FITC_520_/Tm_480_ as a function of the amount of CEA measured on the PAD. The signal of FITC_520_/Tm_480_ increases steadily with the increasing of the CEA concentration. There has a good linear relationship between fluorescent intensity ratio and the concentration of CEA, R, defined as the correlation coefficient of the linear fit, is 0.998, when the concentration range is of 0–100 ng/mL. The limit of detection (LOD) was evaluated using 3 σ/S and found to be 0.89 ng/mL, where σ represents the standard deviation of the blank signal and S is the slope of the calibration plot^28^. In the normal sense, the level of CEA concentration in healthy adult is less than 2.5 ng/mL, whereas the value may exceed 20 ng/mL in case of suffering cancers of the digestive track^29^. Thus, the LOD of the detection system we established can meet the requirements of the real detecting. The inset shows the curve of FITC_520_/Tm_480_ as a function of the amount of CEA, as the concentration is 0–5 ng/mL, it can be seen that the results also exhibited a good linear relationship in the relative low concentration. Note that, Chen and his coworkers reported an assay based on FRET between PFO dots and Au-NPs for CEA detection with the LOD of 2 ng/mL in the range of 0.1–10 ng/mL^30^. Additionally, Liu reported a FRET-based aptasensor using UCNPs as the energy donor and carbon nanoparticles as the acceptor for the detection of CEA with the range of 0.1–40 ng/mL^31^. Compared to the previous reports, the FRET test paper we established had greater detection range, lower detection limit, and is more convenient for CEA detection. The relative standard deviation (RSD) for 5 parallel measurements (intra-assay) of 50 ng/mL CEA is 3.33%. Compared to other reported methods of detection of CEA, the proposed method shows relatively excellent linear range, and detection limit, as shown in Fig. 5^32^,^33^,^34^,^35^,^36^.

**Figure 6:**
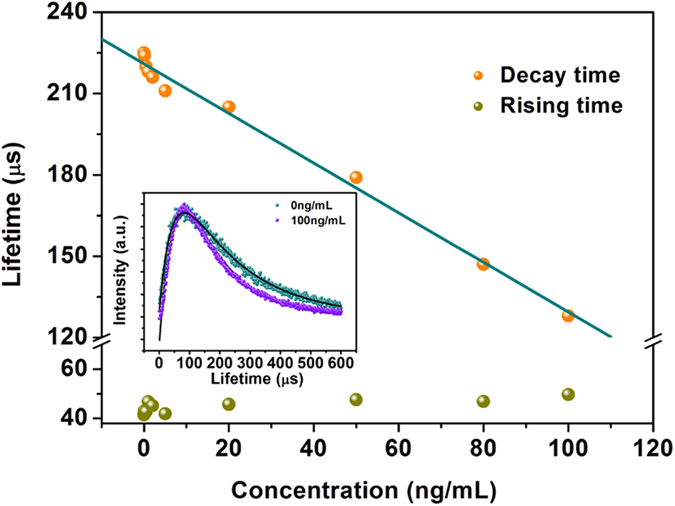
Rising and decay lifetime constants of ^1^G_4_ of Tm^3+^ at different concentrations of CEA (inset: dynamic curves of transition of ^1^G_4_-^3^H_6_ of Tm^3+^ in present and absent of CEA).

To further confirm the FRET process, the dynamic processes of transition of ^1^G_4_-^3^H_6_ of Tm^3+^ with different concentration of CEA were measured. The inset of Fig. 6 shows the upconversion luminescence dynamic curves of transition of ^1^G_4_-^3^H_6_ of Tm^3+^ under 980-nm excitation in absent of and present of 100 ng/mL FITC-CEA. It can be seen that the dynamic curves exhibit the same trends, namely, after an initial rise; the intensity of luminescence reaches a maximum and then decays following an exponential process. The curves can be well fitted by the following equation^37^:


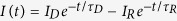


where τ_D_ and τ_R_ represent the decay and rising time constants, respectively, and *I*_D_, *I*_R_ are both the positive constants. Note that the rising time constant τ_R_ is related to the energy transfer rate from Yb^3+^ to Tm^3+^, while τ_D_ depends strongly on the depopulation process of the emission level. The constants of the rising and decay time as a function of the amount of FITC-CEA are shown in Fig. 6. It can be clearly seen that the addition of the acceptor to the donor leads to the decay time of transition of ^1^G_4_-^3^H_6_ of Tm^3+^ reducing from 225 to 128 μs with the increasing concentration of the CEA from 0 to 100 ng/mL, which is a prominent evidence that the non-radiative energy transfer from ^1^G_4_ of Tm^3+^ to the FITC takes place. However, the rising time is almost unchanged with the variation of the concentration. This is owing to that the existence of the FITC has no influence on the energy transfer process from Yb^3+^ to Tm^3+^.

### Anti- interference, stability and reproducibility of PAD

**Figure 7:**
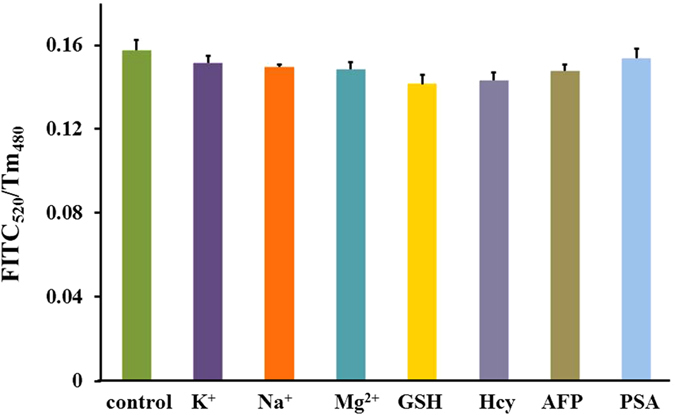
Detection interference for CEA in the absent and present of interference present on PAD, including K^+^, Na^+^, Mg^2+^,GSH, Hcy, AFP, PSA.

Since the test paper based on FRET exhibited the excellent detection performance to CEA, further researches were conducted on it. To monitor the anti- interference performance of the biosensor, some possible interfering agents that potentially co-existed in human serum, such as metal ions, glutathione (GSH), homocysteine (Hcy), and other cancer biomarkers, such as alpha-fetoprotein (AFP), prostate specific antigen (PSA) were selected. 200 ng/mL K^+^, Na^+^, Mg^2+^, GSH, Hcy, AFP, PSA were respectively mixed with 50 ng/mL FITC-CEA and dropped on the test zones, individually, the control was 50 ng/mL FITC-CEA only, then the changes in fluorescence were monitored. From Fig. 7, it can be seen that, compared with the fluorescence change caused by pure CEA, the variation in fluorescence change caused by the interfering substances was less than 12%, even in the condition that the concentration of the interfering agents is 4 times higher than that of CEA, which illustrates the biosensor’s excellent anti-interference performance.

**Figure 8:**
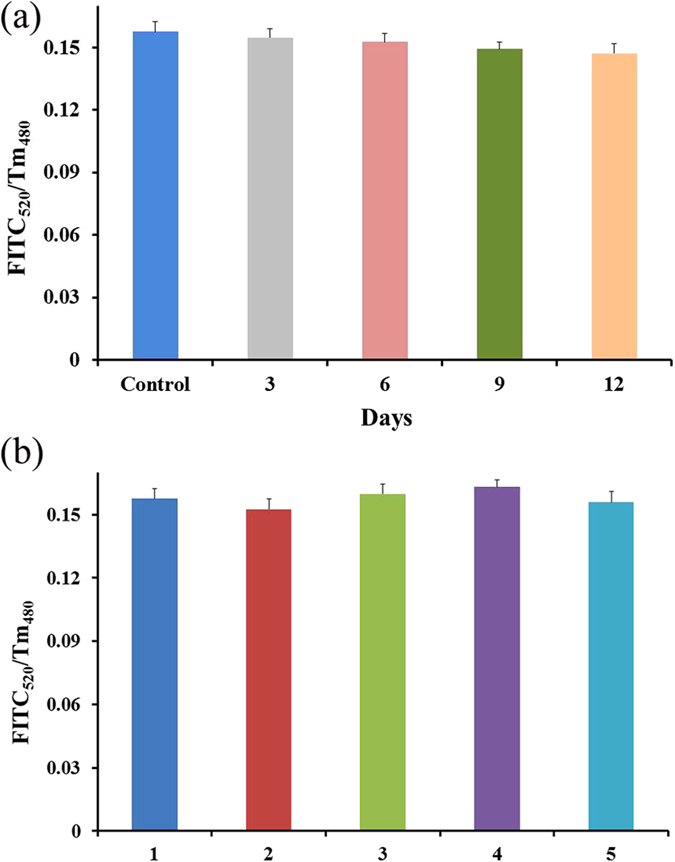
(**a**) Stability of the test paper, the fluorescence change of the test zones which dropped 50 ng/mL FITC-CEA observed every 3 days; (**b**) Repeatability of five different test zones response to 50 ng/mL FITC-CEA.

The stability of the test paper was also tested, as shown in Fig. 8(a), the test paper was stored in 4 ^o^C in the dark condition after prepared. The fluorescence change (FITC_520_/Tm_480_) of different test zones on the same test paper was observed every 3 days (the analyte concentration of FITC-CEA is 50 ng/mL). The response of the test zone measured at 12^th^ day only had a decrease of 6.67% compared to the initial test zone. This indicates the effective retention of the activity of the conjugated CEA antibody. The reproducibility is an important feature to check the reliability of the developed biosensor. Five equally prepared test zones on the same test paper were evaluated by analysis of the same concentration of CEA (50 ng/mL). As shown in Fig. 8(b), five test zones exhibited similar response, which indicates the good repeatability of the test paper. The relative standard deviation (RSD) for 5 parallel measurements (inter-assay) of 50 ng/mL CEA using five different test zones is 4.92%.

### Simultaneous detection of multiple cancer biomarkers

**Figure 9:**
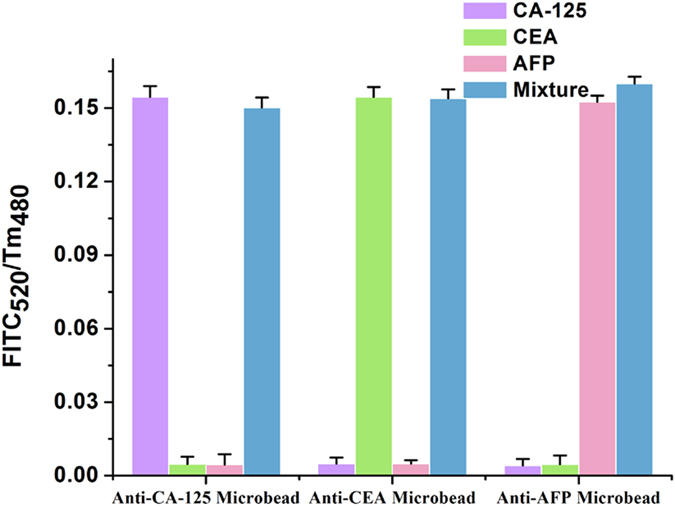
Fluorescence change of each test zone in the presence of CA-125, CEA, AFP and the mixture of the three cancer biomarkers.

As no single cancer biomarker is specific and sensitive enough to meet the strict diagnostic demand, so the analysis of different types of cancer biomarkers offers valuable information for disease diagnostics. In this work, we established a multi-channel test paper for simultaneous detection of multiple cancer biomarkers. First, the UCNPs conjunct with different cancer antibodies were printed on the specific test zone as a fluorescence probe, this was followed by 5 μL 5% BSA solution dropping on the test zone as block, then the paper was dried at 37 ^o^C for 30 min. 50 ng/mL FITC labeled different antigens (CA-125, CEA, AFP) and the mixture of the three antigens were dropped on the specific zone, as shown in Fig. S2. The performance of the multi-channel fluorescence probes with different combinations of three cancer biomarkers was shown in Fig. 9. In the presence of CA-125, CEA or AFP, only anti-CA-125, anti-CEA or anti-AFP test zone showed strong fluorescence change, respectively, while in the presence of the mixture of the three cancer biomarkers, all of the three test zones showed remarkable fluorescence change. These results indicated the high-specificity of the antibodies used in this study which eliminated the cross reaction between antibodies and non-specific analysts, the multi-channel test paper we established can be used for simultaneous analyzing various cancer biomarkers.

### Analysis of clinical serum samples

To monitor the analytical reliability and possible application of the developed paper based biosensor, the test paper was used for the determination of the concentration of CEA in human serum compared to the concentration of CEA in PBS buffer solution under the identical measurement conditions, which was shown in Fig. S7. The curve of the signal of FITC_520_/Tm_480_ exhibited the same trend as that measured in the PBS buffer.

### Analysis in liquid system

The detection was implemented by using PBS solution containing UCNPs instead of the test paper as the biosensor under otherwise identical conditions. Figure S8 showed the variation trend of the signal of FITC_520_/Tm_480_ is same as that measured on the mini test paper zone.

## Conclusions

In conclusion, we have established paper-based analytical devices with UCNPs for sensitive detection of CEA. The features of NIR excitation and anti-Stokes emission of upconversion fluorescence perfectly match the requirements of PAD in clinical analysis. The proposed upconversion PAD offers several notable merits: (1) it is cheap: the paper device can be simply fabricated with normal filter paper; (2) it is highly sensitive: the detection limit is as low as 0.89 ng/mL, which can meet the requirements of the real detecting; (3) it displays good anti-interference, stability and reproducibility. Furthermore, we established a multi-channel test paper for simultaneous detection of multiple cancer biomarkers and obtained positive results. Thus, our efforts suggest that upconversion PAD could be a competent alternative for the development of simple, inexpensive, and sensitive clinical diagnostic technology that may has potential application in biodetection.

## Experimental section

### Synthesis of PEI-modified NaYF_4_:Yb,Tm UCNPs

PEI-modified water-soluble NaYF_4_:Yb,Tm UCNPs were synthesized via a solvothermal method following a procedure reported by Professor Liu^38^. Briefly, 2.5 mmol NaCl, 0.4 g PEI (MW=600, the structural formula was shown in Fig. S1), 0.878 mmol Y(NO_3_)_3_, 0.12 mmol Yb(NO_3_)_3_, and 0.002 mmol Tm(NO_3_)_3_ were dissolved in ethylene glycol (15 mL) under vigorous stirring. After the solution became transparent, NH_4_F (4 mmol) in ethylene glycol (10 mL) was dropwise added to the solution under vigorous stirring. After stirred for another 10 min, the whole mixture solution was transferred into a 25 mL Teflon-lined stainless steel. The autoclave was sealed and heated under 200 °C for 4 h. After the autoclave cooled down to room temperature naturally, NaYF_4_:Yb,Tm UCNPs were collected by centrifugation and washed with deionized water and alcohol three times, then dispersed in carbonate buffer (pH = 9.5) for future use.

### Conjugation of UCNPs with CEA antibody

0.02 ml CEA antibody was added to 5 ml as-prepared UCNPs solution followed by stirring 4 h at the room temperature, then was washed with deionized water to remove the unconjugated antibody, after that, 1% BSA was added as blocking buffer. Finally, the solution was stored at 4 ^o^C in the dark for future use.

### Synthesis of FITC-labeled CEA antigen

Prior to the detection, CEA antigen was labeled with FITC following the well-established protocol^39^. Certain amount of CEA antigen was dissolved in sodium carbonate buffer (pH = 9.5). Meanwhile, 0.2 mg FITC was dissolved in 0.1 mL DMSO. Thereafter, the two solutions were mixed gently and the reaction was allowed to proceed overnight at 4 ^o^C in the dark. The excess FITC was removed via dialysis for 48 h using a membrane of molecular weight cutoff of 14000 Da.

### Synthesis of CuS nanoparticles

Synthesis of CuS nanoparticles was accomplished following a typical hot-injection method by injecting copper precursor into sulfur precursor^40^. All the synthesis was carried out under air-free conditions. A typical synthesis is as follows: Copper precursor was prepared by mixing CuCl (0.01 mol) with a mixture of 4 mL oleic acid and 5 mL oleylamine at 130 °C under continuous stirring. Then, the as-prepared copper precursor was cooled down to room temperature. Sulfur precursor was prepared in a three necked bottle by dissolving sulfur powder (0.01 mol) into 40 mL ODE at 200 °C under mechanical stirring. Subsequently, the sulfur solution was then set to 180 °C, followed by a swift injection of copper precursor and kept at this temperature for 5 to 15 min. After that, the heating metal was removed and cooled down to room temperature. The obtained colloidal solution was then precipitated using excess acetone and recovered by centrifuging the suspension and discarding the supernatant. The precipitation and dispersion was repeated twice, and then the precipitate was dispersed in cyclohexane for future use.

### Characterization

Transmission electron micrographs were obtained with a Hitachi H-800 transmission electron microscope (TEM) operating at an acceleration voltage of 200 kV. The crystalline structure of the samples was characterized by X-ray diffraction (XRD) (RigakuD/max-rA power diffractometer using Cu-KR radiation (λ = 1.54178 Å). Ultraviolet-visible (UV-vis) absorption spectra were measured with a Shimadzu UV-3101PC UV-vis scanning spectrophotometer ranging of 200–1100 nm. The upconversion spectra were measured with a photomultiplier combined with a monochromator for signal collection from 400 nm to 700 nm. A continuous 980-nm diode laser was used to pump the samples to investigate the steady-state spectra. In the measurements of luminescent dynamics, the samples were pumped by a laser-system consisting of a Nd:YAG pumping laser (1064-nm), the third-order Harmonic-Generator (355-nm) and a tunable optical parameter oscillator (OPO, Continuum Precision II 8000). It was with the pulse duration of 10 ns, repetition frequency of 10 Hz and line width of 4–7 cm^−1^.

## Additional Information

**How to cite this article**: Xu, S. *et al.* Paper-based upconversion fluorescence resonance energy transfer biosensor for sensitive detection of multiple cancer biomarkers. *Sci. Rep.* **6**, 23406; doi: 10.1038/srep23406 (2016).

## Supplementary Material

Supplementary Information
