# Supplementary material for: Paper-based upconversion fluorescence resonance energy transfer biosensor for sensitive detection of multiple cancer biomarkers
Source: Sci Rep. 2016 Mar 22;6:23406. doi: 10.1038/srep23406 (PMC4802215; doi:10.1038/srep23406)
Supplement: Supplementary Information [file srep23406-s1.pdf]

Paper-based upconversion fluorescence resonance energy transfer biosensor for sensitive detection of multiple cancer biomarkers

Sai Xu[a,b], Biao Dong[a]*,Donglei Zhou[a], Ze Yin[a], Shaobo Cui[a], Wen Xu[a], Baojiu Chen[b], Hongwei Song[a]*

[a] *State Key Laboratory on Integrated Optoelectronics,*

College of Electronic Science and Engineering, Jilin University, Changchun 130012, People's Republic of China

[b] *Department of Physics, Dalian Maritime University, Dalian 116026, People's Republic of China*

*Corresponding to: Prof. Hongwei Song and Dr. Biao Dong

State Key Laboratory on Integrated Optoelectronics, College of Electronic Science and Engineering, Jilin University

Email: songhw@jlu.edu.cn

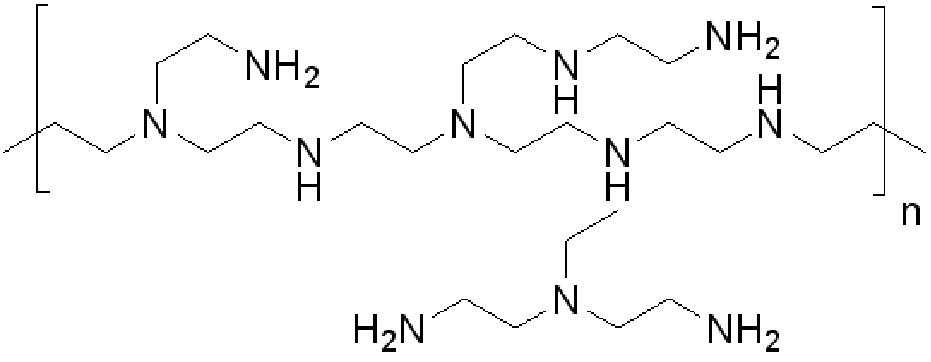

Figure S1 Structural formula of PEI group

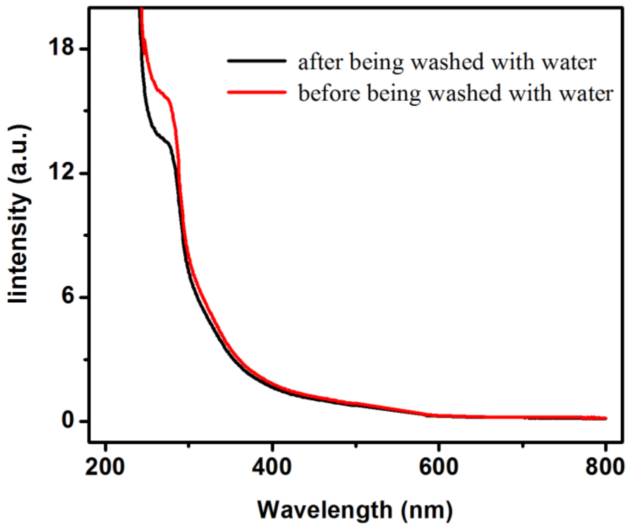

Figure S2 Absorption spectra of the mixed solution before and after being washed with deionized water were measured.

CEA antibody was added to as-prepared UCNPs solution followed by stirring 4h at the room temperature, and then was washed with deionized water to remove the unconjugated antibody. The absorption spectra of the mixed solution before and after being washed with deionized water were measured. From the absorption spectra, the adhesion efficiency of CEA can be calculated as

$$\eta = \frac{A_1 - A_2}{A_1}$$

where A_1 and A_2 represent the intensity of the absorption of the mixed solution before and after being washed with deionized water. From the equation, the adhesion efficiency of CEA is calculated as 87.66%.

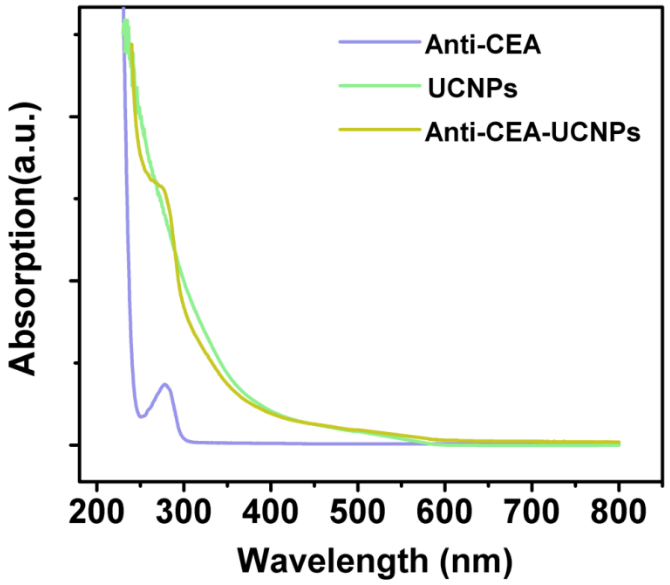

Figure S3 Absorption spectra of Anti-CEA (blue line), UCNPs (green line), and the conjunction of Anti-CEA and UCNPs (yellow line)

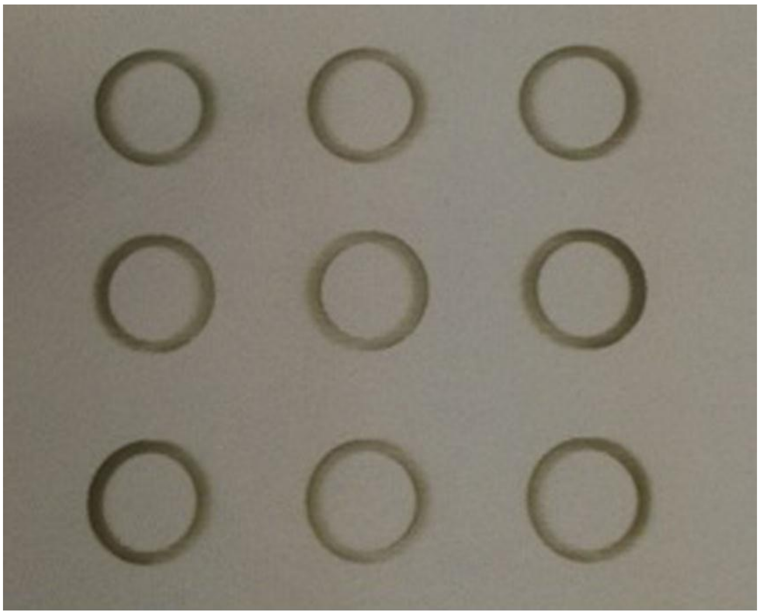

Figure S4 Digital photo of the test paper. Each spot on the paper was isolated with CuS nanoparticles and had a diameter of 1 cm

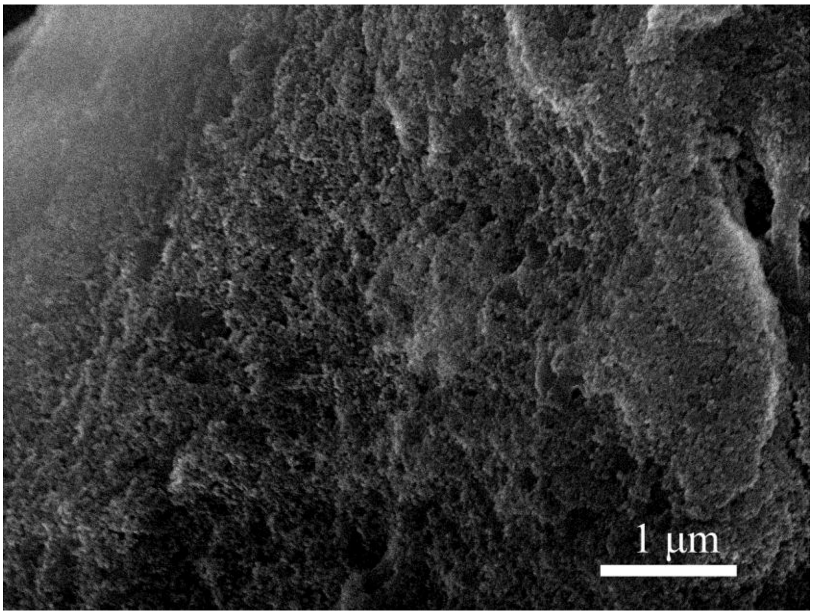

Figure S5 SEM characterization of UCNPs printed on the test paper

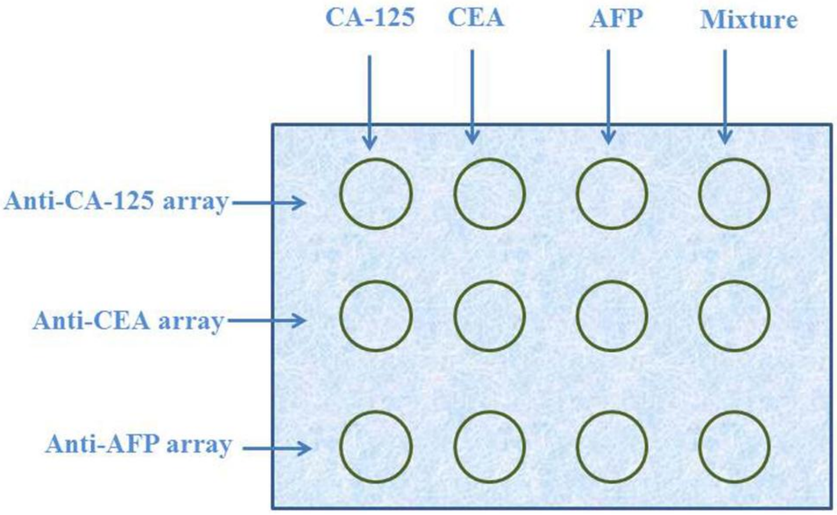

Figure S6 Distribution of the test zone and the analytes on the multi-channel PAD

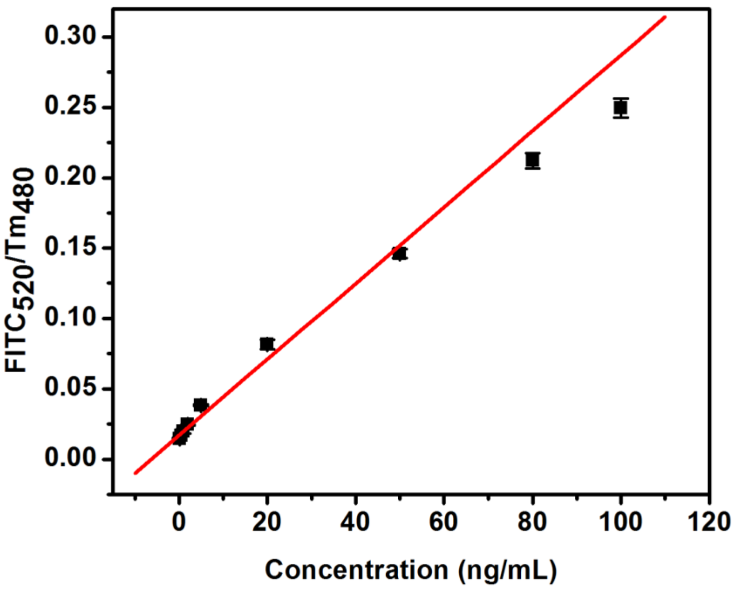

Figure S7 FRET spectra of PAD at different concentrations of CEA in human serum

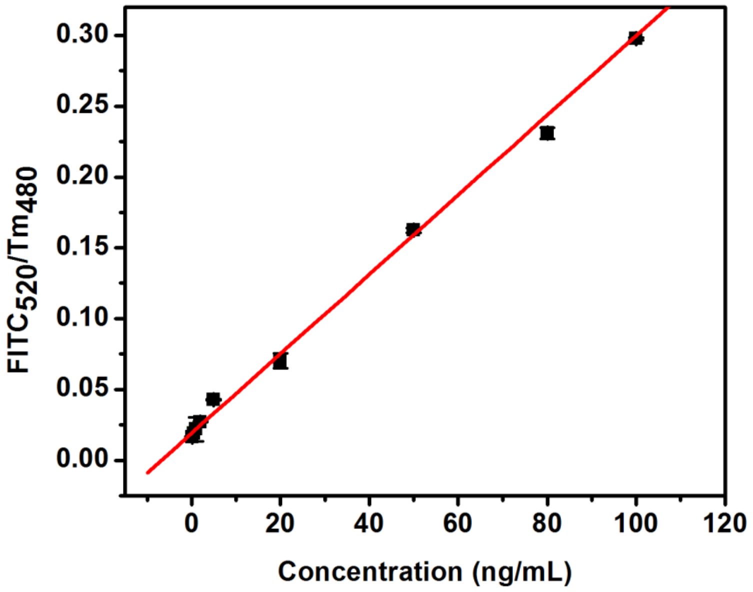

Figure S8 FRET spectra of liquid biosensor at different concentrations of CEA

The liquid biosensor was 3mL PBS buffer solution containing CEA antibody-UCNPs coalition, certain concentration of FITC-CEA was added drop wise to the solution, and then the UC spectra were measured under the same condition as the mini test paper system.
